# Klotho regulates the myogenic response of muscle to mechanical loading and exercise

**DOI:** 10.1113/EP091263

**Published:** 2023-10-20

**Authors:** Eisuke Ochi, Alice Barrington, Michelle Wehling‐Henricks, Marcus Avila, Makoto Kuro‐o, James G. Tidball

**Affiliations:** ^1^ Faculty of Bioscience and Applied Chemistry Hosei University Tokyo Japan; ^2^ Department of Integrative Biology and Physiology University of California Los Angeles CA USA; ^3^ Division of Anti‐Aging Medicine Center for Molecular Medicine Jichi Medical University Tochigi Japan; ^4^ Molecular, Cellular & Integrative Physiology Program University of California Los Angeles CA USA; ^5^ Department of Bioengineering University of California Los Angeles CA USA; ^6^ Department of Pathology and Laboratory Medicine, David Geffen School of Medicine at UCLA University of California Los Angeles CA USA

**Keywords:** exercise, Klotho, myogenesis, satellite cell, skeletal muscle, Wnt

## Abstract

Muscle growth is influenced by changes in the mechanical environment that affect the expression of genes that regulate myogenesis. We tested whether the hormone Klotho could influence the response of muscle to mechanical loading. Applying mechanical loads to myoblasts in vitro increased RNA encoding transcription factors that are expressed in activated myoblasts (*Myod*) and in myogenic cells that have initiated terminal differentiation (*Myog*). However, application of Klotho to myoblasts prevented the loading‐induced activation of *Myog* without affecting loading‐induced activation of *Myod*. This indicates that elevated Klotho inhibits mechanically‐induced differentiation of myogenic cells. Elevated Klotho also reduced the transcription of genes encoding proteins involved in the canonical Wnt pathway or their target genes (*Wnt9a*, *Wnt10a*, *Ccnd1*). Because the canonical Wnt pathway promotes differentiation of myogenic cells, these findings indicate that Klotho inhibits the differentiation of myogenic cells experiencing mechanical loading. We then tested whether these effects of Klotho occurred in muscles of mice experiencing high‐intensity interval training (HIIT) by comparing wild‐type mice and *klotho* transgenic mice. The expression of a *klotho* transgene combined with HIIT synergized to tremendously elevate numbers of Pax7^+^ satellite cells and activated MyoD^+^ cells. However, transgene expression prevented the increase in myogenin^+^ cells caused by HIIT in wild‐type mice. Furthermore, transgene expression diminished the HIIT‐induced activation of the canonical Wnt pathway in Pax7^+^ satellite cells. Collectively, these findings show that Klotho inhibits loading‐ or exercise‐induced activation of muscle differentiation and indicate a new mechanism through which the responses of muscle to the mechanical environment are regulated.

## Abstract (highlights)

- **What is the central question of this study?**
  Does the hormone Klotho affect the myogenic response of muscle cells to mechanical loading or exercise?
- **What is the main finding and its importance?**
  Klotho prevents direct, mechanical activation of genes that regulate muscle differentiation, including genes that encode the myogenic regulatory factor myogenin and proteins in the canonical Wnt signalling pathway. Similarly, elevated levels of *klotho* expression in vivo prevent the exercise‐induced increase in myogenin‐expressing cells and reduce exercise‐induced activation of the Wnt pathway. These findings demonstrate a new mechanism through which the responses of muscle to the mechanical environment are regulated.

## 1 INTRODUCTION

Changes in the mechanical environment of muscle cells can modify the expression of genes that regulate the course of myogenesis. Genes encoding the myogenic regulatory factors myoblast differentiation 1 (MyoD) and myogenin are among the transcripts expressed by developing muscle cells that are sensitive to changes in the mechanical environment. Modifications in the expression of either of these transcription factors can influence myogenesis because MyoD (encoded by *Myod*) plays an important role in activating the expression of genes involved in early stages of the myogenic programme (Weintraub et al., 1991, 1989) and myogenin (encoded by *Myog*) is essential for the terminal differentiation of myogenic cells (Hasty et al., 1993; Nabeshima et al., 1993; Wright et al., 1989). Application of cyclic strains to mouse C2C12 myoblasts in vitro produced significant increases in the expression of both *Myod* and *Myog* (Chandran et al., 2007; Moustogiannis et al., 2022), showing that mechanical activation of those genes does not require the presence of non‐muscle cells and suggesting that mechanical loading directly promotes myogenesis.

Many of the changes in transcriptional activity of muscle cells that are caused by direct, mechanical stimulation in vitro also occur in muscle in vivo during increased loading and exercise, indicating that part of the response of muscle to exercise may result from direct mechanical stimulation of muscle cells. For example, muscle overload caused by exercise or by synergist ablation increased the number of myogenic cells expressing MyoD (Ishido et al., 2004; Tamaki et al., 2000) and myogenin in muscle (Ishido et al., 2004). In addition, mechanical loading in vitro or exercise in vivo activates a population of myogenic cells, called satellite cells, that express the transcription factor Pax7 (Kook et al., 2008; Mackay et al., 2011; Seale et al., 2000). Some of those activated satellite cells then proliferate and proceed to differentiate to express MyoD and myogenin, after which the cells can contribute to increases in muscle mass and increases in muscle fibre size (Cornelison & Wold, 1997; Schiaffino et al., 1976; Smith et al., 1994). Other activated satellite cells return to a quiescent state and restore the reserve population of myogenic cells that reside in muscle (Baroffio et al., 1996; Beauchamp et al., 1999; Olguin & Olwin, 2004; Yoshida et al., 1998). Thus, exercise or mechanical loading of muscle can promote myogenesis by expanding the numbers of Pax7^+^ satellite cells and by increasing the numbers of myogenic cells expressing MyoD and myogenin that progress through the course of muscle differentiation during muscle growth.

The canonical Wnt signalling pathway is one of multiple pathways that mediate the response of muscle to exercise. Wnt proteins are secreted growth factors that bind receptors in the Frizzled (Fzd) family, leading to activation of an intracellular, canonical signalling pathway that culminates with the translocation of β‐catenin to the cell nucleus, where it participates in the transcriptional activation of specific target genes (Girardi & Le Grand, 2018; Nusse, 2008). Typically, those target genes influence cell proliferation, differentiation and migration. Exercise or increased muscle loading in vivo produced changes in activation of the canonical Wnt pathway that were associated with effects on myogenesis and muscle growth. For example, increased muscle loading caused by synergist ablation in mice caused large increases in muscle mass that were accompanied by nuclear targeting of β‐catenin and elevated expression of the Wnt target genes *Myc* (encoding c‐myc) and *Ccnd1* (encoding cyclin D1) (Armstrong & Esser, 2005). Similarly, treadmill running increased activation of the canonical Wnt pathway (Aschenbach et al., 2006; Polesskaya et al., 2003; Rochat et al., 2004; van der Velden et al., 2006) that was associated with elevated expression of Wnt ligands in muscle and increased *Myod* and *Myog* expression (Fujimaki et al., 2014). Furthermore, the findings that increased signalling through the canonical Wnt pathway in injured muscle accelerated muscle fibre formation, followed by a reduction in satellite cell numbers, and that activation of the canonical Wnt pathway increased satellite cell differentiation and muscle fibre growth following injury (Bernardi et al., 2011; Han et al., 2011) suggest that the increased activation of Wnt signalling in exercised muscle may directly contribute to changes in satellite cell numbers and increased muscle growth. However, we do not know whether activating the expression of Wnt ligands or Wnt target genes in exercised muscle results from the direct, mechanical stimulation of myogenic cells, independent of the presence of non‐muscle cells.

The influences of mechanical loading on myogenesis could potentially be influenced by Klotho, an anti‐ageing protein. Klotho is expressed in many tissues, including skeletal muscle, as a secreted isoform (s‐Klotho) that functions as a hormone or as an alternatively spliced, transmembrane protein (m‐Klotho) (Kurosu et al., 2005; Li et al., 2004; Matsumura et al., 1998; Shiraki‐Iida et al., 1998). The extracellular domain of m‐Klotho can be cleaved and released (Chen et al., 2007; Imura et al., 2004), to function along with s‐Klotho as a hormone. Several observations indicate that Klotho can affect myogenesis. First, reductions in Klotho that result from genetic deletion, natural ageing or disease are associated with reductions of muscle mass (Kurosu et al., 2005; Wehling‐Henricks et al., 2016), suggesting that Klotho acts either directly or indirectly on muscle to affect myogenesis. At least some of Klotho's effects on muscle occur via direct actions; Klotho can act directly on myogenic cells in vitro by increasing their proliferation and protein content and by affecting the expression of myogenic regulatory factors (Wehling‐Henricks et al., 2016; Welc et al., 2020). In addition, overexpression of *klotho* affects early post‐natal myogenesis by increasing satellite cell numbers and influencing the epigenetic regulation of genes that control myogenesis (McKee et al., 2022). Similarly, overexpression of *klotho* in the *mdx* mouse model of Duchenne muscular dystrophy increased satellite cell numbers and prevented muscle atrophy that occurs in late stages of the disease (Wehling‐Henricks et al., 2016) and elevated Klotho levels increased numbers of satellite cells and accelerated muscle growth following acute injury (Welc et al., 2020) and improved regeneration of injured, aged muscle (Sahu et al., 2018).

At least some of the influences of Klotho on myogenesis are attributable to its effects on Wnt signalling. In a cell free system, Klotho can bind to several Wnt ligands, including Wnt1, Wnt3a, Wnt4, Wnt5a and Wnt7a (Liu et al., 2007; Zhou et al., 2013), which is sufficient to inhibit the activity of at least Wnt3a (Liu et al., 2007). Application of exogenous Klotho to isolated muscle fibres also reduced Wnt signalling, which was attributed to Klotho binding to extracellular Wnt (Ahrens et al., 2018). However, Klotho can also reduce Wnt signalling by down‐regulating the expression of Wnt ligands and receptors. Treatment of myoblasts with Klotho in vitro reduced expression of *Wnt4*, *Wnt9a*, *Wnt10a*, *Fzd3* and *Fzd9*, which was associated with increased H3K27 methylation in the promoter region of each of those genes (McKee et al., 2022). Similarly, elevated expression of *klotho* in vivo reduced the expression of Wnt target genes (*Ccnd1* and *Myc*) and inhibited canonical Wnt signalling in muscle following acute injury (Welc et al., 2020). Collectively, these observations indicate that Klotho's effects on myogenesis are mediated in part through its negative regulation of the canonical Wnt pathway.

In the current investigation, we tested the hypothesis that elevated levels of Klotho can modulate the response of muscle to direct, mechanical loading and exercise. We first tested whether application of Klotho to myoblasts in vitro influences the mechanically‐induced expression of key transcription factors and Wnt family genes that regulate myogenesis. We then assayed whether expression of a *klotho* transgene affects changes in the number of myogenic cells that express myogenic transcription factors in the muscles of mice that have experienced high‐intensity interval training (HIIT) and assayed whether those effects are accompanied by changes in activation of canonical Wnt signalling in myogenic cells.

## 2 METHODS

### 2.1 Ethical approval

All mice were handled according to guidelines provided by the Chancellor's Animal Research Committee at the University of California, Los Angeles (UCLA) (Animal Welfare Assurance number A3196). This investigation complies with the standards for reporting animal experiments (Grundy, 2015) and complies with the animal ethics required by *Experimental Physiology*.

### 2.2 Mice

C57BL/6J mice were purchased from The Jackson Laboratory (Bar Harbor, ME, USA) and bred and housed in a specific pathogen‐free vivarium at UCLA in which they were provided food and water ad libitum. The production of transgenic mice that overexpressed *klotho* (EFmKL46) has been previously described (Kuro‐o et al., 1997). Expression of the *klotho* transgene is controlled by the elongation factor 1a promoter that causes approximately two‐fold increase in Klotho in circulation (Kurosu et al., 2005). The transgene encodes the entire open reading frame of Klotho. Mice carrying the *klotho* transgene were crossed onto the C57BL/6 background for a minimum of six generations (Welc et al., 2020). Experimental mice were killed by inhalation of isoflurane, after which the tibialis anterior (TA), gastrocnemius (Gastr) and quadriceps femoris (Quad) muscles were weighed, rapidly frozen and stored in liquid‐nitrogen‐cooled isopentane for histological analysis or frozen in liquid nitrogen for qPCR analysis.

### 2.3 Cyclic mechanical loading of myoblasts

**FIGURE 1:**
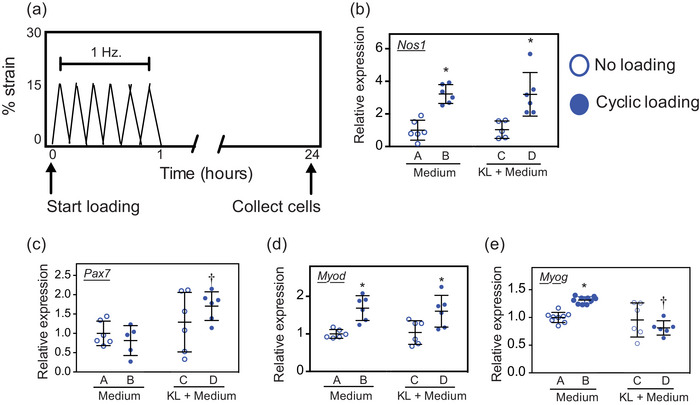
Klotho stimulation and mechanical stimulation interact to affect the expression of *Pax7* and *Myog*. (a) Schematic diagram of mechanical loading applied to myoblasts in vitro. Silastic membranes to which myoblasts adhered were subjected to a 15% cyclic strain at 1 Hz for 1 h. Cells were collected 23 h after the end of the 1 h of cyclic loading for analysis of RNA. (b) qPCR data showing increased expression *Nos1* in myoblasts experiencing loading, in the absence (*P* = 0.0012) or presence (*P* = 0.0023) of exogenous Klotho. Data were normalized to expression in ‘no loading’, medium‐only myoblasts, set at ‘1’. Sample sizes for groups A–D: 5, 6, 5, 6. (c) qPCR data showing that cyclic loading and exogenous Klotho synergized to increase expression of *Pax7* (*P* = 0.0130). Sample sizes for groups A–D: 6, 5, 6, 6. (d) qPCR data showing increased expression *Myod* in myoblasts experiencing loading, independent of the addition of exogenous Klotho (*P*‐values in Table 2). Sample sizes for groups A–D: 6, 6, 6, 6. (e) qPCR data showing increased expression of *Myog* in myoblasts experiencing loading, and the prevention of loading‐induced *Myog* expression caused by the addition of exogenous Klotho (*P*‐values in Table 2). Sample sizes for groups A–D: 9, 9, 6, 6. * Significantly different from unloaded group under same Klotho stimulation conditions at *P* < 0.05. † Significantly different from no exogenous Klotho group under same loading conditions at *P* < 0.05.

Myoblasts (C2C12; ATCC, Manassas, VA, USA; RRID:CVCL_0188) were plated at 15,600 cells/cm^2^ in six‐well, Bioflex cell culture plates (Flexcell International Corp., Burlington, NC, USA) with flexible, Silastic‐membrane well‐bottoms, coated with collagen type 1. Cells were cultured for 48 h in complete medium (Dulbecco's modified Eagle's medium (DMEM) containing 4.5 g/l d‐glucose and 10% fetal bovine serum (FBS), 100 U/ml penicillin and 100 μg/ml streptomycin at 37°C and 5% CO_2_. Cells reached ∼70% confluence at 24 h and were then transferred to medium that did not contain FBS but contained either heparin (10 μg/ml) or heparin + Klotho (1 μg/ml; R&D Systems, Minneapolis, MN, USA). The recombinant Klotho used in the investigation is the full‐length protein minus the signal sequence and a portion of the small intracellular domain of the protein. The recombinant protein added to the culture medium functions as soluble Klotho. The culture plates were then transferred to a cell loading apparatus (FX‐3000, Flexcell International Corp.). Cyclic, radial strains were applied to the flexible bottoms of the wells and to the attached cells by cyclic application of negative pressure. The mean strain of the loaded membrane was 15%, which is within the physiological range experienced by skeletal muscle. The strain cycle consisted of 1 h of cyclic, sinusoidal strains applied at 1.0 Hz followed by 23 h without applied strain (Figure 1a). Muscle cells in no‐loading wells were treated identically, except no strain was applied. After the 23‐h rest period, the muscle cells were washed twice with phosphate buffered saline (PBS) and collected in Trizol (Thermo Fisher Scientific, Waltham, MA, USA) for RNA analysis.

### 2.4 High‐intensity interval training

Mice initiated an 8‐week HIIT protocol at ∼11 months of age, with tissue collecting occurring at ∼13 months of age. The training was modelled on a previously reported HIIT protocol designed to progressively increase the amount of work performed each week (Goh et al., 2019). HIIT sessions were completed on an Exer‐6M Treadmill (Columbus Instruments, Columbus, OH, USA). Prior to HIIT, mice were acclimated to the treadmill during week 0 by running three times at a speed of 8 m/min for 15 min with no incline. Mice were subjected to three sessions of HIIT per week for 8 weeks. During the first 3 weeks of HIIT, the angle of inclination of the treadmill increased 5° weekly from 10° at week 1 to 20° at week 3. During weeks 3–8, the treadmill incline was maintained at 20°. Each session consisted of a 5‐min warm‐up at 8 m/min, followed by eight exercise intervals at the prescribed speed and angle of inclination for 3–5 min, with 1‐min breaks at 8 m/min between intervals. During weeks 1 through 4, treadmill speed increased over the eight sessions from 15 to 16 m/min. During weeks 5, 6, 7 and 8, treadmill speed increased from 15 to 17 m/min, 15 to 18 m/min, 15 to 19 m/min, and 15 to 20 m/min, respectively. Mice that did not stay on the treadmill were shocked by an electric stimulus from the shock grid beneath the treadmill at a current of 4 mA and a frequency of 5 Hz. Mice that did not quickly resume running after landing on the shock grid were manually guided back on the treadmill. Mice that did not resume running after manual assistance were excluded from the study.

### 2.5 RNA isolation and qPCR

RNA was isolated from 50 mg of frozen TA muscle following homogenization in Trizol. We selected TA muscle for analysis because previous investigations showed that HIIT increases TA muscle growth (Goh et al., 2019; Seldeen et al., 2018) and showed that Klotho affects TA muscle growth following injury or in diseased muscle (Wehling‐Henricks et al., 2016; Welc et al., 2020). RNA was extracted with chloroform and precipitated with isopropanol. RNA was then DNase‐treated and purified with RNeasy Mini Kit (Qiagen, Hilden, Germany) according to manufacturer's protocol. Total RNA was quantified by spectrophotometry (Beckman Coulter, Brea, CA, USA) at 260 nm absorbance. RNA samples had a concentration greater than or equal to 0.2 μg/μl and absorbance ratio of 1.8 or higher. The RNA was electrophoresed on agarose gels and its quality assessed by 28S and 18S ribosomal RNA integrity (Wehling‐Henricks et al., 2016). RNA samples (2 μg) were reverse transcribed with Super Script Reverse Transcriptase II using oligo dTs to prime extension (Thermo Fisher Scientific) to produce cDNA. Expression of selected transcripts was assayed using SYBR green qPCR Supermix according to the manufacturer's protocol (Bio‐Rad Laboratories, Hercules, CA, USA) and an iCycler thermocycler system equipped with iQ5 optical system software (Bio‐Rad). Established guidelines for experimental design, data normalization and data analysis for qPCR were used to maximize the rigor of quantifying the relative levels of mRNA (Nolan et al., 2006). The relative expression of transcripts of interest was normalized to the reference genes *Srp14* and *Hagh* for muscle regeneration. The normalization factor for each sample was calculated by geometric averaging of the *C*_t_ values of reference genes. Expression for each gene in control samples was set to 1 and the other values were scaled to the control. Primers used for qPCR are listed in Table 1.

**TABLE 1:** Primer sequences.

Gene	Forward	Reverse
*Axin2*	GACGCACTGACCGACGATTC	CTGCGATGCATCTCTCTCTGG
*Ccnd1*	CGAGGAGCTGCTGCAAATG	GGGTTGGAAATGAACTTCACATC
*Eff1a1*	TTGGTTCAAGGGATGGAAAG	AGC AAA GGT AAC CAC CAT GC
*Fgf23*	GCACTGCTAGAGCCTATCCG	GCACTGTAGATGGTCTGATGG
*Hagh*	CACCACTCACCACCACTGG	ACACTGAGAGACCCCACCTG
*klotho*	GTCTCGGGAACCACCAAAAG	CTATGCCACTCGAAACCGTC
*Myod*	GAGCGCATCTCCACAGACAG	AAATCGCATTGGGGTTTGAG
*Myog*	CCAGTACATTGAGCGCCTAC	ACCGAACTCCAGTGCATTGC
*Nos1*	GGACCTGGGCTAAGAAGTC	GGAGCTTCTGCCACATAAGTG
*Pax7*	CTCAGTGAGTTCGATTAGCCG	AGACGGTTCCCTTTGTCGC
*Rnps1*	AGGCTCACCAGGAATGTGAC	CTTGGCCATCAATTTGTCCT
*Srp14*	AGAGCGAGCAGTTCCTGAC	CGGTGCTGATCTTCCTTTTC
*Tgfb1*	CTCCACCTGCAAGACCAT	CTTAGTTTGGACAGGATCTGG
*Vangl2*	CCAAGTCCGTCCTGGCCAAG	GCTCATGCTCGGCTTCCTCG
*Wnt4*	GAGAAGTTTGACGGTGCCAC	GTCCTCATCTGTATGTGGCTTG
*Wnt9*	GACTTCCACAACAACCTCGTG	AGGAGCCAGACACACCATG
*Wnt10a*	CGAATGAGACTCCACAACAACCG	CGTGGCATTTGCACTTACGC

RNA from C2C12 cells was extracted and isolated with chloroform extraction and isopropyl alcohol precipitation followed by clean‐up using RNAeasy spin columns (Qiagen) and concentrator kit (Zymo Research, Irvine, CA, USA). *Eff1a1* and *Srp14* were used as reference genes.

### 2.6 Pax7 immunohistochemistry

Frozen sections 10 μm thick were fixed with 4% paraformaldehyde for 10 min and then immersed in antigen retrieval buffer (10 mM sodium citrate, 0.05% Tween 20, pH 6.0) for 40 min at 95°C. Sections were then incubated in 0.3% hydrogen peroxide in PBS for 10 min to quench endogenous peroxidase activity. A mouse‐on‐mouse immunohistochemistry kit (M.O.M. kit, Vector Laboratories, Newark, CA, USA) was used to treat the sections with blocking buffer for 60 min, followed by immunolabelling with affinity purified mouse anti‐Pax7 (1:500; Developmental Studies Hybridoma Bank, Iowa City, IA, USA) overnight at 4°C. Sections were then labelled with biotin‐conjugated anti‐mouse IgG (1:250) for 30 min and incubated with ABC reagent from the M.O.M. kit. Staining was visualized with the peroxidase substrate 3‐amino‐9‐ethylcarbazole (AEC; Vector Laboratories), yielding a red reaction product.

Pax7^+^ cells were quantified in sections by observations through an Olympus BH50 microscope using a ×40 objective lens, with the sections overlaid with a calibrated, 10 × 10 eyepiece micrometer grid. All Pax7^+^ cells were counted in one, entire, midbelly cross section of each muscle analysed. The number of cells per unit volume was determined by counting the number of grid intercepts overlying tissue in the field to determine tissue area and then multiplying that value by the section thickness (10 μm). The numbers of satellite cells were then expressed as the number of cells per unit volume in the section.

### 2.7 MyoD immunohistochemistry

Sections were dried for 30 min, fixed in cold acetone for 10 min and then air dried for 10 min. After immersion in PBS, sections were incubated in 0.3% hydrogen peroxide in PBS for 10 min to quench endogenous peroxidase activity, washed in PBS and then incubated in M.O.M. kit blocking buffer for 60 min. The sections were subsequently washed with PBS and incubated in goat anti‐mouse Fab fragment (1:10) for 1 h. After washing with PBS, sections were incubated overnight at 4°C in mouse anti‐MyoD (1:50; BD Pharmingen, San Diego, CA, USA; cat no. 554130; RRID:AB_395255) diluted in protein diluent from the M.O.M. kit. Sections were then washed in PBS and incubated in M.O.M. kit secondary anti‐mouse IgG (1:200) for 30 min. The sections were further treated and analysed as described above for Pax7 immunohistochemistry.

### 2.8 Pax7/β–catenin double‐labelling

Frozen sections cut at 10 μm thickness were fixed, quenched and blocked as described above for Pax7 immunohistochemistry. Sections were then co‐labelled with mouse anti‐Pax7 (1:500) and rabbit antibodies to active, non‐phosphorylated (Ser45) β‐catenin (1:1500; Cell Signaling Technology, Danvers, MA, USA, cat. no. 19807T; RRID:AB_2650576) overnight in a humidified chamber at 4°C. Sections were subsequently washed and incubated for 30 min with horse anti‐mouse Dylight‐594 (1:200; Vector Laboratories; RRID:AB_2336412) and horse anti‐rabbit Dylight‐488 (1:100; RRID:AB_2336403). Sections were mounted with Prolong Gold antifade mountant containing DNA stain 4′,6‐diamidino‐2‐phenylindole (DAPI; cat. no. P36931; Thermo Fisher Scientific). Cell counts were performed on one, entire, midbelly cross section of each muscle analysed. Cells were first assessed for whether they were Pax7 positive and then assessed for whether they were also active β‐catenin positive. Data are expressed as the percentage of total Pax7^+^ satellite cells that also expressed active β‐catenin (β‐catenin^+^ Pax7^+^/ total Pax7^+^). Cell counts were performed on a Leica TCS‐SP5 confocal microscope.

### 2.9 Muscle fibre cross‐sectional area

Cross sections of 10 μm thickness were taken from the mid‐belly of TA muscles frozen in isopentane. Sections were incubated in haematoxylin for 10 min, and then rinsed with distilled water. Stained sections were imaged using a digital imaging system (Bioquant, Nashville, TN, USA) and an Olympus BH2 microscope equipped with Nomarski optics. The area and minimum Feret diameter of 500 randomly selected fibres per cross section were measured using ImageJ. The classification for large or small fibres was determined by setting 3 standard deviations from the mean cross‐sectional area (CSA) for the control group at each time point as previously described (White et al., 2009).

### 2.10 Assay for myonuclear number

Sections were fixed and blocked following the same procedure as for MyoD labelling. Sections were incubated overnight at 4°C in mouse anti‐dystrophin (1:30; Novacastra, Newcastle upon Tyne, UK; NCL‐DYS2) diluted in protein diluent from the M.O.M. kit. Following primary antibody incubation, sections were washed in PBS and incubated in M.O.M. kit secondary anti‐mouse IgG (1:200) for 30 min. The sections were then incubated with ABC reagent from the M.O.M. kit. After rinsing in water for 1.5 min, they were incubated in haematoxylin for 3 min, and then rinsed with water. Dystrophin staining was visualized with the AEC peroxidase substrate (Vector Laboratories), yielding a red reaction product. Myonuclei were then quantified in sections by observations through an Olympus BH50 microscope. The number of myonuclei per fibre was determined by counting the number of haematoxylin‐stained nuclei located deep to the dystrophin^+^ lamina in a total of 500 fibres per section.

### 2.11 Statistical analysis

Statistical outliers were identified using Grubbs's outlier test (*P* < 0.05) before data analysis. Statistical significance was calculated using an unpaired Student's *t‐*test when two groups were compared or ordinary one‐way ANOVA with Tukey's multiple comparison test to determine differences among multiple groups. Differences with a *P‐*value <0.05 were considered statistically significant. Statistical analysis was performed using GraphPad Prism (GraphPad Software, Boston, MA, USA). All graphs display means ± standard deviation, with individual data points plotted. All *P*‐values for all groups that were compared are provided in Table 2, sorted according to the figure panel in which the data appear.

**TABLE 2:** *P*‐values for data group comparisons, sorted according to figures.

Groups	Figure 1b	Figure 1c	Figure 1d	Figure 1e			
A vs. B	0.0012	0.9264	0.0062	0.0009			
A vs. C	>0.9999	0.7523	0.9970	0.9468			
A vs. D	0.0130	0.1030	0.0171	0.1264			
B vs. C	0.0220	0.4206	0.0097	0.0008			
B vs. D	>0.9999	0.0384	0.9676	<0.0001			
C vs. D	0.0023	0.4898	0.0264	0.4019			
	Figure 2a	Figure 2b	Figure 2c	Figure 2d	Figure 2e	Figure 2f	Figure 2g
A vs. B	0.0010	0.9997	0.5827	0.0086	0.9976	0.7393	0.6841
A vs. C	>0.9999	<0.0001	0.3859	0.5340	0.9921	0.2314	>0.9999
A vs. D	>0.9999	<0.0001	0.4877	0.0033	0.0196	0.0859	0.6092
B vs. C	0.0044	<0.0001	0.9565	<0.0001	0.9656	0.7501	0.6254
B vs. D	0.0046	<0.0001	0.9875	<0.0001	0.0130	0.4078	0.9992
C vs. D	>0.999	0.2582	0.9875	0.5416	0.1355	0.9347	0.5465
	Figure 3b	Figure 3c	Figure 3e				
A vs. B	>0.9999	>0.9999	<0.0001				
A vs. C	<0.0001	0.8267	0.0123				
A vs. D	<0.0001	>0.9999	0.3298				
B vs. C	<0.0001	0.7023	<0.0001				
B vs. D	<0.0001	0.9986	<0.0001				
C vs. D	0.0775	0.8811	0.0001				
	Figure 4c	Figure 4f	Figure 4i				
A vs. B	0.5199	0.7046	0.0008				
A vs. C	0.8021	0.9958	0.9894				
A vs. D	0.0006	0.0494	0.9995				
B vs. C	0.1342	0.5696	0.0016				
B vs. D	0.0170	0.3925	0.0003				
C vs. D	<0.0001	0.0295	0.9952				
	Figure 5a	Figure 5b	Figure 5c	Figure 5d	Figure 5e	Figure 5f	Figure 5 g
A vs. B	0.0037	0.9865	0.1829	0.9992	0.0230	0.9880	0.0319
A vs. C	<0.0001	0.1869	0.0097	0.0181	0.0025	>0.9999	0.0001
A vs. D	<0.0001	0.3337	0.0002	0.0021	0.0010	0.4261	<0.0001
B vs. C	0.0018	0.3173	0.4734	0.0239	0.7466	0.9868	0.0076
B vs. D	0.0002	0.5363	0.0294	0.0029	0.6987	0.2556	0.0969
C vs. D	0.9481	0.9305	0.4917	0.9263	>0.9999	0.4325	0.3877
	Figure 6c	Figure 6d	Figure 6e	Figure 6f	Figure 6j		
A vs. B	0.9968	0.9970	0.2748	0.8656	0.9014		
A vs. C	0.1928	0.2084	†	†	0.9840		
A vs. D	0.0827	0.1058	†	†	0.3853		
B vs. C	0.2712	0.2891	†	†	0.9919		
B vs. D	0.0527	0.1590	†	†	0.7306		
C vs. D	0.9442	0.9865	0.5987	0.6038	0.6214		
E vs. F	†	†	0.4980	0.1512	†		

Groups are identified by designations on histograms in the figures. † Groups that were not compared statistically.

## 3 RESULTS

### 3.1 Klotho stimulation and mechanical stimulation interact to affect the expression of genes that regulate myogenesis

We tested whether Klotho treatments could modify the transcriptional response of muscle to mechanical loading by subjecting myoblasts in vitro to 1 h of cyclic stretching at 1 Hz and then collecting the cells 23 h later to assay for changes in the expression of transcription factors (Figure 1a). Identical populations of myoblasts experienced no stretching or cyclic stretching in the presence of Klotho‐supplemented medium. First, we confirmed that our loading protocol was sufficient to activate mechanically‐sensitive genes by assaying for changes in *Nos1* expression (Figure 1b; *P*‐values provided in Table 2), which we previously showed to be expressed at elevated levels in myoblasts subjected to stretch in vitro (Tidball et al., 1998). Although neither stretch alone (*P* = 0.9264) nor Klotho stimulation alone (*P* = 0.7524) affected *Pax7* expression, co‐stimulation of myogenic cells with both stretch and Klotho produced a more than 70% increase in *Pax7* expression, compared to stretch alone (*P* = 0.0384) (Figure 1c). Expression of the myogenic transcription factor *Myod* was elevated by stretch in the presence (*P* = 0.0264) or absence (*P* = 0.0062) of Klotho stimulation, but *Myod* expression was not affected by Klotho in either unstretched (*P* = 0.9970) or stretched (*P* = 0.9676) myoblasts (Figure 1d). Finally, we observed that mechanical loading elevated the expression of *Myog* (*P* = 0.0009) but stimulation of myoblasts with Klotho prevented the mechanical activation of *Myog* expression (*P* < 0.0001), without affecting the expression of *Myog* in non‐stretched myoblasts (*P* = 0.9468) (Figure 1e). Thus, Klotho stimulation can synergize with mechanical stimulation to increase expression of myogenic transcription factors (*Pax7*), or block the mechanical activation of myogenic transcription factors (*Myog*) or show no influence on the mechanical activation of myogenic transcription factors (*Myod*).

**FIGURE 2:**
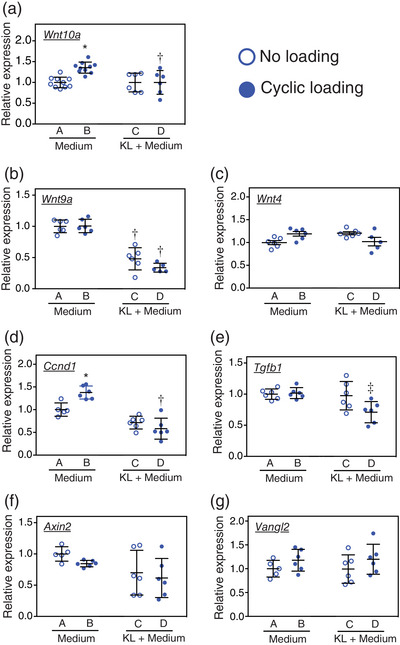
Exogenous Klotho prevents the increased expression of *Wnt10a* and *Ccnd1* that is caused by mechanical stimulation. (a) qPCR data show that in the absence of Klotho stimulation, mechanical loading increases *Wnt10a* expression (*P* = 0.0010), but the loading‐induced increase does not occur in the presence of exogenous Klotho. Sample sizes for groups A–D: 10, 10, 6, 6. (b) qPCR data show that mechanical loading does not increase *Wnt9a* expression (*P* = 0.9997), but exogenous Klotho reduces *Wnt9a* independent from the application of mechanical loading (*P*‐values in Table 2). Sample sizes for groups A–D: 6, 6, 6, 6. (c) *Wnt4* expression is unaffected by the presence of exogenous Klotho or the application of mechanical loading (*P*‐values in Table 2). Sample sizes for groups A–D: 6, 6, 6, 6. (d) qPCR data show that in the absence of Klotho stimulation, mechanical loading increases *Ccnd1* expression (*P* = 0.0086), but exogenous Klotho prevents the loading‐induced increase (*P* < 0.0001). Sample sizes for groups A–D: 6, 6, 6, 6. (e) *Tgfb1* expression is reduced by the combined application of exogenous Klotho and mechanical loading (*P* = 0.0196), but not by application of either Klotho (*P* = 0.9921) or loading alone (*P* = 0.9976). Sample sizes for groups A–D: 6, 6, 6, 6. (f) Application of Klotho does not affect *Axin2* expression in either ‘no loading’ or ‘cyclic loading’ conditions (*P*‐values in Table 2). Sample sizes for groups A–D: 6, 6, 6, 6. (g) *Vangl2* expression is unaffected by the presence of exogenous Klotho or the application of mechanical loading (*P*‐values in Table 2). * Significantly different from unloaded group under same Klotho stimulation conditions at *P* < 0.05. Sample sizes for groups A–D: 6, 6, 6, 6. † Significantly different from no exogenous Klotho group under same loading conditions at *P* < 0.05. ‡ Significantly different from both the no exogenous Klotho group under same loading conditions and from the unloaded group under same Klotho stimulation conditions at *P* < 0.05.

Our previous work showed that Klotho stimulation of myoblasts in vitro influences the expression of genes in the Wnt family that play important roles in regulating muscle differentiation. In particular, the expression of *Wnt4*, *Wnt9a* and *Wnt10a* was reduced by treating myoblasts with Klotho (McKee et al., 2022). We assayed in vitro whether Klotho stimulation affected the response of myoblasts to mechanical loading and found the response varied between different Wnt family genes. Loading in the absence of Klotho stimulation caused a significant, 36% elevation in *Wnt10a* expression (*P* = 0.0010) that was blocked by co‐stimulation with Klotho (Figure 2a), although stimulation with Klotho alone, in the absence of mechanical stimulation, did not affect *Wnt10a* expression compared to non‐stimulated myoblasts (*P* > 0.9999) (Figure 2a). Because elevated Klotho stimulation did not affect *Wnt10a* expression in non‐loaded myogenic cells, the data show that the effect of Klotho stimulation is to block the mechanically‐induced activation of *Wnt10a* expression, rather than reducing activation of *Wnt10a* expression independent of loading. In contrast, *Wnt9a* expression was not affected by stretch, in the presence (*P* = 0.2582) or absence (*P* = 0.9997) of Klotho stimulation (Figure 2b). However, Klotho stimulation significantly reduced *Wnt9a* expression by ∼50–65%, independent of the occurrence (*P* < 0.0001) or absence (*P* < 0.0001) of mechanical loading. Unlike either *Wnt10a* or *Wnt9a*, neither mechanical stretch (*P* = 0.5827) nor Klotho stimulation (*P* = 0.3859), applied separately or together (*P* = 0.4877), affected *Wnt4* expression (Figure 2c).

We also assayed whether the effects of mechanical stretch and Klotho stimulation were manifested in the expression of Wnt target genes. Similar to our findings concerning the effects on Wnt ligand expression, we found that Klotho stimulation blocked the mechanical activation of Wnt target genes (*Ccnd1; P* < 0.0001; Figure 2d) or reduced the expression of Wnt target genes that did not exhibit mechanical activation (*Tgfb1*; *P* < 0.0130; Figure 2e) or showed no significant effect on Wnt target gene expression, independent of mechanical stimulation (*Axin2*; Figure 2f; *P*‐values provided in Table 2). We also found no influence of mechanical stretch or Klotho stimulation on *Vangl2* expression (Figure 2g; *P*‐values provided in Table 2), which functions in non‐canonical Wnt signalling. Collectively, these observations show that the direct stimulation of myoblasts with Klotho can block the mechanical activation of select target genes involved in regulating myogenesis.

### 3.2 Elevated *klotho* expression reduces activation of Wnt signalling in myogenic cells in muscles of mice experiencing HIIT

**FIGURE 3:**
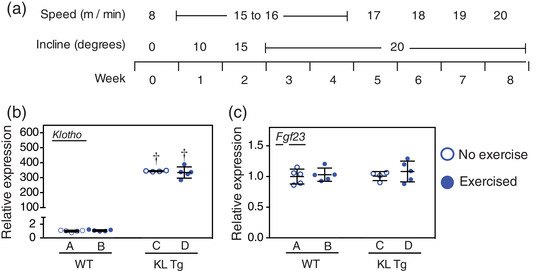
HIIT does not affect expression of endogenous or transgenic *klotho* or *Fgf23* in muscle. (a) Schematic diagram of the HIIT protocol used for mouse exercise. Following a period of acclimating to treadmill running during week zero, the treadmill speed and the angle of incline of the treadmill were increased progressively over the 8‐week HIIT period. (b, c) qPCR data show that HIIT did not affect expression of *klotho* or *Fgf23* in either wild‐type (WT) or KL Tg mice (*P*‐values in Table 2). Sample sizes for groups A–D: 5, 5, 4, 5. † Significantly different from wild‐type mice under same exercise conditions at *P* < 0.05.

**FIGURE 4:**
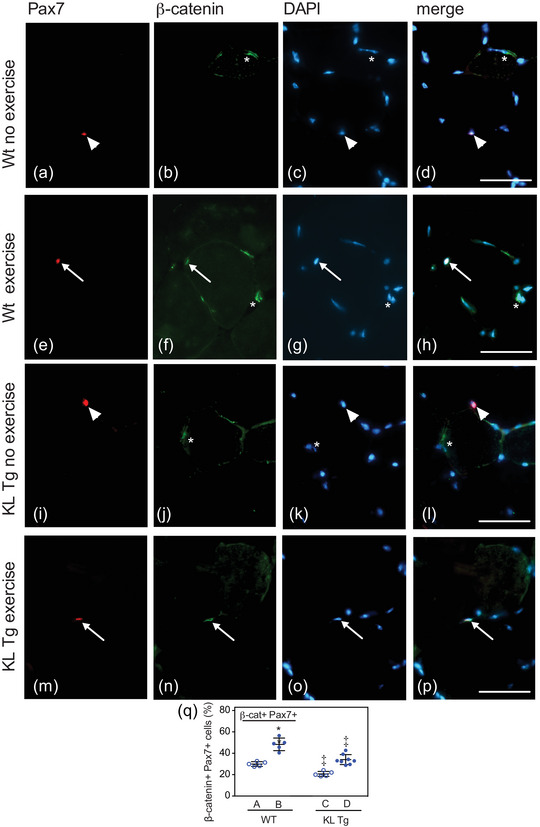
Expression of a *klotho* transgene reduces the exercise‐induced activation of the canonical Wnt pathway in satellite cells. (a–p) Micrographs of TA muscle cross sections that were triple‐labelled with anti‐Pax7 (red), anti‐activated β‐catenin (green), and DAPI (blue). Merged images combining all three wavelengths are shown as ‘merge’ (d, h, I, p). Nuclei that are Pax7^+^/β‐catenin^−^ are indicated with an arrowhead. Nuclei that are Pax7^+^/β‐catenin^+^ are indicated with an arrow. Nuclei that are Pax7^−^/β‐catenin^+^ are indicated with an asterisk. Scale bars: 50 μm. (q) Quantification of the percentages of total Pax7^+^ cells that contained active β‐catenin shows that exercise increased the proportion of Pax7^+^ cells in which active β‐catenin was detected, but the magnitude of exercised‐induced activation was reduced by *klotho* transgene expression (*P*‐values in Table 2). * Significantly different from no exercise group of mice of the same genotype at *P* < 0.05. ‡ Significantly different from wild‐type mice in the same treatment group at *P* < 0.05. Sample sizes for groups A–D: 5, 5, 5, 8.

Because the findings reported above show that Klotho blocks the mechanical activation of some Wnt ligands and Wnt target genes in a simplified, in vitro environment, we tested whether elevated *klotho* expression affected the mechanical activation of Wnt signalling in the complex in vivo environment during exercise. Using a modification of an established HIIT technique that has been shown to affect TA muscle growth and adaptation (Goh et al., 2019) (Figure 3a), we first confirmed that HIIT in wild‐type or KL transgenic (KL Tg) mice did not affect expression of *klotho* or *Fgf23*, which can influence Klotho signalling (Figure 3b,c; *P*‐values provided in Table 2). We then assayed whether HIIT or elevated *klotho* expression applied separately or together affected activation of Wnt signalling by determining the proportion of Pax7^+^ myogenic cells in TA muscles that contained β‐catenin that was dephosphorylated on serine 45, which is required for β‐catenin activation and for canonical Wnt signalling. HIIT caused a significant, 42% increase in the proportion of Pax7^+^ cells that contained detectible levels of active β‐catenin (*P* < 0.0001) (Figure 4a–h,q), establishing that exercise produced activation of canonical Wnt signalling in satellite cells. However, elevated *klotho* expression reduced the proportion of satellite cells that exhibited detectible β‐catenin activation in HIIT TA muscles by 38% (*P* < 0.0001) (Figure 4i–p, q).

### 3.3 Elevated *klotho* expression affects the shift in myogenic cell phenotype that occurs during HIIT in vivo

**FIGURE 5:**
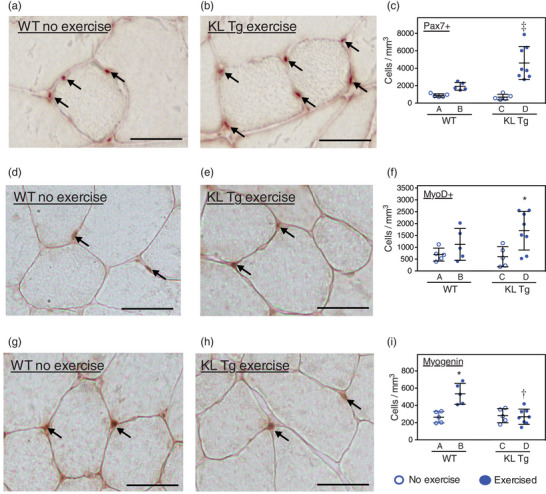
Expression of a *klotho* transgene modified the myogenic response of muscle to exercise. (a, b) Representative micrographs of cross sections of TA muscles showing cells immunolabelled with anti‐Pax7 (arrows). (c) Quantification of the numbers of Pax7^+^ cells per volume of muscle shows a synergistic interaction between HIIT and *klotho* transgene expression that increases numbers of Pax7^+^ cells (*P*‐values in Table 2). Sample sizes for groups A–D: 5, 5, 5, 8. (d, e) Representative micrographs of cross sections of TA muscles showing cells immunolabelled with anti‐MyoD (arrows). (f) Quantification of the numbers of MyoD^+^ cells per volume of muscle shows a synergistic interaction between HIIT and *klotho* transgene expression that increases numbers of MyoD^+^ cells (*P*‐values in Table 2). Sample sizes for groups A–D: 5, 5, 5, 8. (g, h) Representative micrographs of cross sections of TA muscles showing cells immunolabelled with anti‐myogenin (arrows). (i) Quantification of the numbers of myogenin^+^ cells per volume of muscle shows that the increase in myogenin^+^ cells that is caused by HIIT (*P* = 0.0008) is prevented by expression of the *klotho* transgene (*P* = 0.0003). Sample sizes for groups A–D: 5, 5, 5, 8. Scale bars: 50 μm. * Significantly different from no exercise group of mice of the same genotype at *P* < 0.05. †Significantly different from wild‐type mice under same exercise conditions at *P* < 0.05. ‡Significantly different from both the group of wild‐type mice under the same exercise conditions and from the no exercise group of mice of the same genotype at *P* < 0.05.

Our findings showed that Klotho treatments influenced the response of myogenic cells to mechanical stimulation in vitro by affecting the expression of genes that regulate myogenesis (Figure 1). We assessed whether those in vitro effects were also manifest in vivo by assaying whether increased expression of *klotho* modulated the influence of HIIT on the numbers of myogenic cells that expressed Pax7, MyoD or myogenin in vivo. Although HIIT alone did not affect the numbers of Pax7^+^ (*P* = 0.5199) or MyoD^+^ cells (*P* = 0.7046) in wild‐type mice and elevated Klotho alone did not affect the numbers of Pax7^+^ or MyoD^+^ cells in wild‐type or transgenic mice (Figure 5a–f; *P*‐values provided in Table 2), the combination of HIIT and elevated Klotho synergized to produce large increases in the numbers of both of Pax7^+^ (*P* = 0.0006) or MyoD^+^ cells (*P* = 0.0494) (Figure 5a–f). Together, HIIT and elevated Klotho increased the numbers of Pax7^+^ cells by 524% and MyoD^+^ cells by 246%, relative to non‐exercised, wild‐type mice. In contrast, HIIT alone produced a significant 203% increase in the number of myogenin^+^ cells (*P* = 0.0008), although Klotho alone had no effect, relative to non‐exercised wild‐type mice (*P* = 0.9894) (Figure 5g–i). In addition, overexpression of *klotho* in mice experiencing HITT completely blocked the increase in myogenin^+^ cells that occurred in non‐transgenic mice experiencing HIIT (*P* = 0.0003). Thus, the general effect of elevated Klotho on the myogenic response to HIIT is to prevent the increase in myogenic cells that have begun terminal differentiation and expanding the population of activated myogenic cells that have not begun to terminally differentiate.

### 3.4 Increased myogenic cell numbers by HIIT and Klotho is not associated with increased muscle growth

**FIGURE 6:**
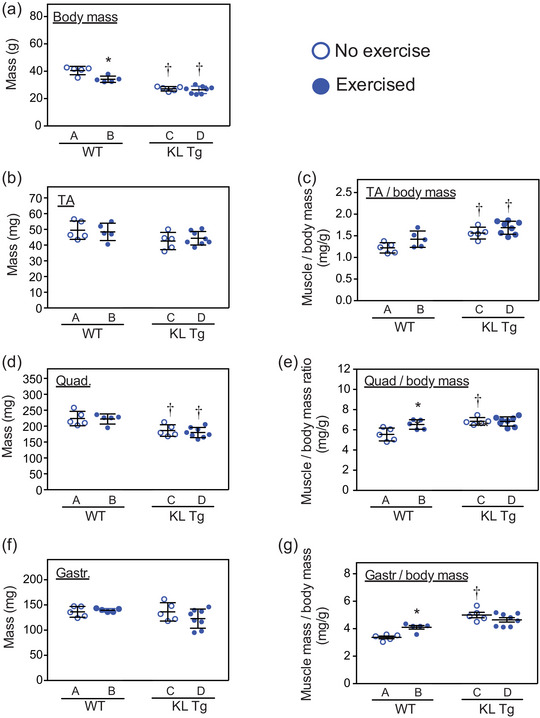
Neither HIIT or *klotho* transgene expression, applied separately or together, increased body or muscle mass. (a) Exercise reduced body mass of WT mice (*P* = 0.0037) and *klotho* transgene expression decreased mass in both the ‘no exercise’ and ‘exercised’ groups (*P* < 0.0001 and *P* = 0.0002, respectively). Sample sizes for groups A–D: 5, 5, 5, 8. (b, c) TA masses did not differ between any groups analysed (b) (*P*‐values in Table 2) although TA mass normalized to body mass was greater in KL Tg mice (c) (*P*‐values in Table 2), attributable the lesser body mass of KL Tg mice. Sample sizes for groups A–D: 5, 5, 5, 8. (d) *klotho* transgene expression decreased Quad mass in both the ‘no exercise’ and ‘exercised’ groups (*P* = 0.0230 and *P* = 0.0025, respectively). Sample sizes for groups A–D: 5, 5, 5, 8. (e) Either exercise or *klotho* transgene expression increased Quad mass normalized to body mass (*P* = 0.0181 and *P* = 0.0029, respectively). Sample sizes for groups A–D: 5, 5, 5, 8. (f) Gastr masses did not differ between any groups analysed (*P*‐values in Table 2). Sample sizes for groups A–D: 5, 5, 5, 8. (g) Either exercise or *klotho* transgene expression increased Gastr mass normalized to body mass (*P* = 0.0319 and *P* = 0.0001, respectively). Sample sizes for groups A–D: 5, 5, 5, 8.

Previous investigations have demonstrated that changes in the numbers of Pax7^+^ cells in muscle correspond to changes in muscle fibre growth during development, disease, ageing or in response to increased exercise or loading (Brack et al., 2005; Egner et al., 2016; Fry et al., 2015; Murach et al., 2017; Rostami et al., 2022; Wehling‐Henricks et al., 2018; White et al., 2010) although in some experimental models, large reductions in Pax7^+^ cell numbers did not affect the hypertrophic response of muscles to increased loading (McCarthy et al., 2011). We tested whether the expansion of Pax7^+^ cells and MyoD^+^ cells in exercised TA muscles of KL Tg mice corresponded with changes in TA muscle mass or fibre size. However, we found that increased *klotho* expression reduced total body mass in both non‐exercised (*P* < 0.0001) and exercised mice (*P* = 0.0002) (Figure 6a). This was attributable to reduction in adiposity caused by the transgene, as previously reported (Rao et al., 2019). Neither HIIT nor increased Klotho influenced TA mass (Figure 6b) and the elevation of TA/body mass ratio in KL Tg mice was therefore a result of the reduction in body mass caused by the transgene (Figure 6c; *P*‐values provided in Table 2). Similarly, the increase in numbers of Pax7^+^ and MyoD^+^ cells in KL Tg mice experiencing HIIT was not accompanied with increases in the masses of quadriceps or gastrocnemius muscles (Figure 6d–g). On the contrary, the masses of quadriceps muscles of KL Tg HIIT mice were less than the masses of wild‐type HIIT mice (*P* = 0.0029) (Figure 6d).

**FIGURE 7:**
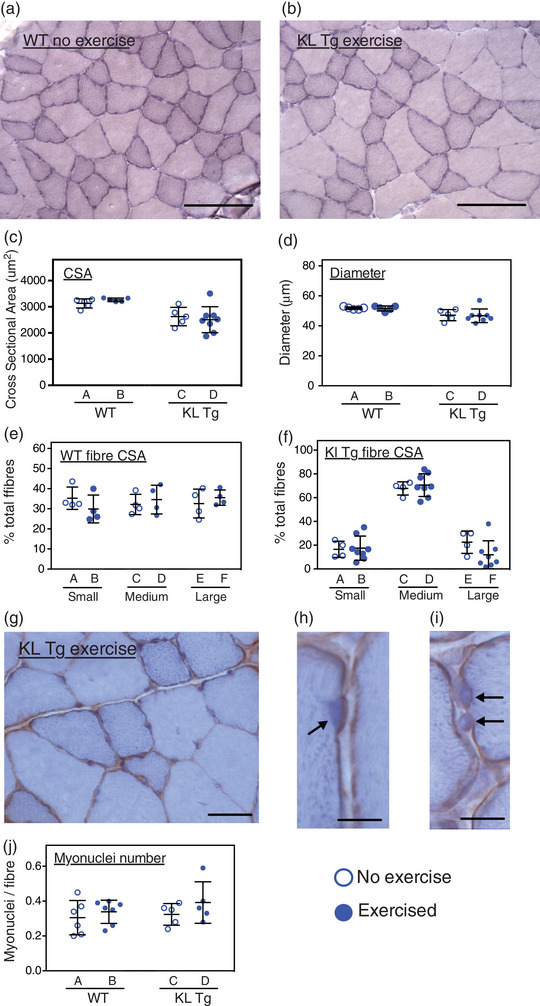
*Klotho* transgene expression did not promote muscle fibre growth or increase myonuclear number in HIIT mice. (a, b) Micrographs of cross sections of TA muscles showing relative distribution of muscle fibre CSAs. Scale bars: 70 μm. (c, d) *Kotho* transgene expression and HIIT, applied either together or separately, did not affect muscle fibre CSA or minimum Feret diameter (*P*‐values in Table 2). Sample sizes for groups A–D: 5, 5, 5, 8. (e, f) HIIT did not affect the distribution of muscle fibre CSAs in either WT (sample sizes for groups A–F: 4, 4, 4, 4, 4, 4) or Klotho transgenic mice (sample sizes for groups A–F: 4, 8, 4, 8, 4, 8) (*P*‐values in Table 2). (g) Representative micrograph of cross section of TA muscle immunolabelled with anti‐dystrophin (brown) and stained with haematoxylin (blue) to identify location of nuclei within or outside the muscle fibres. Scale bar: 40 μm. (h) Myonucleus (arrow) stained dark blue with haematoxylin and located subjacent to the dystrophin lamina lying within the muscle fibre, deep to the cell membrane. Scale bar: 8 μm. (i) Nuclei (arrows) lying outside of the muscle fibre, external to the dystrophin lamina. Scale bar: 12 μm. (j) Quantification of the number of myonuclei per muscle fibre observed in cross sections shows no differences between any of the four treatment groups (*P*‐values in Table 2). Sample sizes for groups A–D: 6, 7, 5.

Because changes in muscle fibre growth are not always reflected by changes in muscle mass (Egner et al., 2016), we assayed whether HIIT or elevated Klotho affected fibre cross section or the distribution of fibre sizes. Consistent with the absence of increased muscle mass in mice experiencing HIIT or Klotho overexpression or both HIIT and elevated Klotho, we found no increase in muscle CSA or minimum Feret diameter in groups of mice experiencing HIIT or increased Klotho or both HIIT and elevated Klotho (Figure 7a–d; *P*‐values provided in Table 2). HIIT also had no effect on the distribution of muscle fibre sizes in either wild‐type or *klotho* transgenic mice (Figure 7e–f).

### 3.5 Elevated Klotho and HIIT do not affect myonuclear number

Our finding that the numbers of myogenin^+^ cells were greatly elevated by exercise in muscles of wild‐type mice but not in exercised, KL Tg mice could feasibly occur if myogenic cells in KL Tg mice exited the stage of development at which myogenin was down‐regulated sooner than wild‐type myogenic cells exited that stage. Down‐regulation of myogenin would be followed by fusion of the myogenic cells with fully differentiated muscle fibres or other terminally differentiating myogenic cells (Andres & Walsh, 1996). We tested that possibility by assaying whether the lower numbers of myogenin^+^ cells in KL Tg HIIT mice were accompanied by elevated numbers of myonuclei in muscle fibres, compared to wild‐type HIIT mice. Our findings show no differences in the number of myonuclei/fibre between any of the treatment groups (Figure 7g–j; *P*‐values provided in Table 2), indicating that the lower numbers of myogenin^+^ cells in the KL Tg HIIT muscles were not attributable to their earlier differentiation and fusion.

## 4 DISCUSSION

The most fundamental finding in our current investigation is that Klotho can prevent the direct, mechanical activation of genes that regulate muscle differentiation. In particular, we observed that mechanical loads applied to myoblasts in vitro increased expression of *Myod*, *Myog*, *Wnt10a* and *Ccnd1* but the application of exogenous Klotho prevented the increased expression of *Myog*, *Wnt10a* and *Ccnd1*. Because exogenous Klotho did not affect the expression of any of those transcripts in myoblasts that did not undergo loading, our findings show that Klotho specifically reduces the mechanical activation of those genes, rather than simply reducing their expression that occurs independent of loading. Our results also predict that exogenous Klotho can inhibit the differentiation of myogenic cells that occurs during increased loading. Ablation of the mechanically activated transcription of *Myog*, *Wnt10a* and *Ccnd1* and the loading‐independent inhibition of *Wnt9a* expression are consistent with Klotho inhibition of differentiation during mechanical loading because myogenin expression is essential for muscle differentiation (Hasty et al., 1993; Nabeshima et al., 1993; Wright et al., 1989) and elevated Wnt signalling is associated with differentiation of myogenic cells (Brack et al., 2008, 2007). In addition, we observed a synergy between mechanical loading and Klotho treatments on increasing the expression of *Pax7*, which encodes a protein that can slow muscle differentiation, which is attributable in part to inhibition of MyoD activation of *Myog* expression (Olguin & Olwin, 2004; Olguin et al. 2007; Zammit et al., 2006; von Maltzahn et al., 2013).

The effects of *klotho* transgene expression in mice experiencing HIIT resembled the effects of Klotho stimulation on mechanically loaded myogenic cells in vitro. For example, we found that elevated *klotho* expression inhibited the exercise‐induced activation of the canonical Wnt pathway in Pax7^+^ cells. This outcome is consistent with Klotho inhibition of the canonical Wnt pathway that occurs in satellite cells on isolated muscle fibres in vitro (Ahrens et al., 2018) and following acute injury of adult muscle (Welc et al., 2020) and in developing, neonatal muscle (McKee et al., 2022). We also found the combination of *klotho* overexpression and HIIT synergized to produce large increases in the number of Pax7^+^ cells, similar to the synergistic effects of mechanical loading and Klotho on *Pax7* expression in myogenic cells in vitro. Likewise, we observed that *klotho* overexpression prevented the HIIT‐induced increase in myogenin^+^ cells in TA muscles, resembling the Klotho‐mediated ablation of elevated *Myog* expression in mechanically stimulated myogenic cells in vitro. Although our data do not prove unequivocally that the changes in myogenesis in HIIT mice that were produced by elevated *klotho* expression resulted from inhibition of Wnt signalling, previous findings by other investigators strongly support that interpretation. For example, treating myoblasts with Wnt3a reduced the expression of *Pax7* (Hulin et al., 2016; Zhuang et al., 2014), overexpression of Wnt4, Wnt6, Wnt7a, or Wnt9a accelerated differentiation of myogenic cells in vitro (Tanaka et al., 2011) and pharmacological activation of Wnt signalling in muscle accelerated muscle differentiation (Rochat et al., 2004; van der Velden et al., 2006), potentially leading to reduction of satellite cell numbers (Brack et al., 2008). Each of these effects of elevated Wnt signalling in muscle is the inverse of the treatment effects we observed in vivo when inhibiting canonical Wnt signalling by expressing a *klotho* transgene in mice experiencing HIIT.

Although *klotho* transgene expression during HIIT produced large increases in the numbers of Pax7^+^ cells and MyoD^+^ cells, the increase in myogenic cells was not accompanied by increased muscle fibre growth or muscle mass in our investigation. This was contrary to our initial expectation because several, previous studies reported that increases in satellite cell numbers that occur in muscle during exercise or increased loading were accompanied by muscle hypertrophy (Egner et al., 2016; Fujimaki et al., 2014; Masschelein et al., 2020; McCarthy et al., 2011; Rostami et al., 2022; Shefer et al., 2010). However, other findings indicate that increasing satellite cell numbers per se is likely not the important correlate for exercise‐induced muscle hypertrophy; instead, fusion of myogenic cells that are derived from satellite cells to muscle fibres may be the important determinant. For example, genetic ablation of *Mymk*, which encodes the myomaker protein that is necessary for myogenic cell fusion with muscle fibres (Millay et al., 2013), prevented increases in muscle fibre CSA in mouse plantaris muscles subjected to compensatory hypertrophy (Goh & Millay, 2017) and in hindlimb muscles of mice experiencing 8 weeks of HIIT (Goh et al., 2019). However, not all increases in myogenic cell fusion with muscle fibres that are caused by exercise are sufficient to increase fibre CSA. After 8 weeks of voluntary treadmill running without resistance, soleus muscles of mice showed increased fusion of myogenic cells with muscle fibres without producing an increase in muscle fibre size (Masschelein et al., 2020). Conversely, rats experiencing 6 weeks of voluntary treadmill running showed increased CSA of muscle fibres in the vastus lateralis, without an increase in the number of myonuclei per fibre (Smith & Merry, 2012). Notably, other previous investigations have reported some muscle hypertrophy without the recruitment of new myonuclei to muscle fibres (Petrella et al., 2006, 2008). Some of the reported differences in the relationship between myonuclear accretion and muscle fibre growth in response to exercise may be attributable to differences in the exercise protocol or the muscle assayed or the species that was studied. However, the observation that increases in myogenic cell fusion with muscle fibres during exercise occur weeks before measurable increases in muscle fibre CSA (Goh et al., 2019) also shows that the duration of the exercise protocol and the time of muscle sampling can have a large influence in assessing relationships between muscle cell fusion and muscle fibre growth.

In our investigation, we observed that HIIT and *klotho* transgene expression, either separately or together, did not affect numbers of myonuclei, despite the large increases in Pax7^+^ and MyoD^+^ cells in KL Tg mice experiencing HIIT. This indicates that the expansion of myogenic cell numbers without a concomitant increase in fusion with muscle fibres is insufficient to increase fibre CSA, at least in the exercise model that we investigated. Notably, we did not observe hypertrophy in any of the treatment groups although the HIIT protocol that we used was very similar to a HIIT protocol used previously that produced increased muscle fibre CSA and increased numbers of myonuclei in wild‐type mice (Goh et al., 2019). However, our 8‐week HIIT experiments on wild‐type mice in the present investigation used ∼13‐month‐old mice (corresponding to ∼46‐year‐old humans) while the previous 8‐week HIIT experiments used ∼8‐month‐old mice (corresponding to ∼35‐year‐old humans) (Goh et al., 2019). Because the mice in our investigation were older, reducing the workload was necessary to enable them to complete the HIIT; during weeks 4 through 8 of treadmill training, mice in our investigation ran on a 20° incline, although the previous HIIT protocol used 25° (Goh et al., 2019). Thus, the workload in our investigation may have been insufficient to drive increases in muscle fibre size, similar to previous findings in which 8 weeks of free, voluntary treadmill running did not produce muscle fibre hypertrophy although 8 weeks of voluntary treadmill running with extra resistance provided by a weighted running wheel produced hypertrophy (Masschelein et al., 2020).

A potentially interesting but unanswered question in our investigation is: what is the fate of the large number of Pax7^+^ and MyoD^+^ cells in the muscles of KL Tg mice at the end of 8 weeks of HIIT? *klotho* transgene expression combined with HIIT produced a 664% increase in Pax7^+^ cells and a 283% increase in MyoD^+^ cells compared to non‐exercised, transgenic mice. As a consequence, more than 85% of all Pax7^+^ satellite cells that were present in transgenic muscles after HIIT were generated while the mice were exercise training. During post‐natal development in mice, satellite cell number/muscle fibre decline drastically, falling over 70% between 7 days and 21 days post‐natal, and the numbers then remain unchanged until at least 10 months old, which was the oldest age sampled in the study (White et al., 2010). Thus, the combination of elevated Klotho levels and 8 weeks of HIIT returns satellite cell numbers in adult muscle to levels that exceed young, post‐natal mice. If those numbers persist long‐term in muscle, that could feasibly restore a youthful, regenerative capacity in old muscle, which is partially limited by the loss of satellite cells during old age (Brack et al., 2008). In addition, the large number of satellite cells generated during HIIT in KL Tg mice may be qualitatively different from those that were generated during development in the absence of exercise. Discoveries in the most recent few years have shown that skeletal muscle retains an epigenetic memory that persists in detrained muscle even after the muscle has returned to pre‐exercise mass (Seaborne, Strauss, Cocks, Shepherd, O'Brien, van Someren, Bell, Murgatroyd, Morton, Stewart, Mein, et al., 2018; Seaborne, Strauss, Cocks, Shepherd, O'Brien, van Someren, Bell, Murgatroyd, Morton, Stewart, & Sharples, 2018; Turner et al., 2019; Wen et al., 2021). In the likely event that satellite cell DNA also experiences long term epigenetic modifications during exercise, the large number of satellite cells that are generated in muscles during HIIT in the presence of elevated Klotho may have significant and possibly beneficial modifications to their future, transcriptional responses to muscle injury, adaptation, disease or ageing.

## 5 CONCLUSIONS

Our investigation shows that mechanical loads applied directly to myoblasts can activate the expression of transcription factors that regulate muscle differentiation and genes involved in canonical Wnt signalling, which also promotes muscle differentiation. However, application of exogenous Klotho prevents the loading‐induced expression of those genes. Similarly, expression of a *klotho* transgene in mice experiencing HIIT increased the numbers of myogenic cells that expressed Pax7 and MyoD but prevented exercise‐induced increases in myogenin^+^ cells, consistent with Klotho inhibition of differentiation of activated satellite cells during exercise. That inhibition of differentiation was accompanied by a Klotho‐induced reduction in activation of canonical Wnt signalling in Pax7^+^ satellite cells. Although these effects of elevated Klotho on the response of myogenic cells to exercise produced tremendous increases in the numbers of activated satellite cells in exercised muscle, they did not increase muscle growth during exercise. This failure for the large increase in satellite cell numbers to contribute to exercise‐induced hypertrophy appears attributable to the inability of the activated cells to proceed to terminal differentiation and fusion with muscle fibres in the presence of elevated levels of Klotho.

## AUTHOR CONTRIBUTIONS

All authors contributed to the conception, design or performance of the research. Eisuke Ochi, Alice Barrington, Michelle Wehling‐Henricks, Marcus Avila and James G. Tidball performed the experiments. Makoto Kuro‐o generated the Klotho transgenic mouse line that is used in the investigation. Eisuke Ochi and James G. Tidball analysed the data and wrote the manuscript. All authors have read and approved the final version of this manuscript and agree to be accountable for all aspects of the work in ensuring that questions related to the accuracy or integrity of any part of the work are appropriately investigated and resolved. All persons designated as authors qualify for authorship, and all those who qualify for authorship are listed.

## CONFLICT OF INTEREST

The authors declare that they have no conflicts of interest. The content of this article is solely the responsibility of the authors and does not necessarily represent the official views of the National Institutes of Health.

## Data Availability

The data sets analysed in the investigation are available from the corresponding author on reasonable request.
